# In vitro cytotoxicity analysis of Zizyphus spina-christi stem bark extract on human cancer cell lines

**DOI:** 10.6026/97320630017583

**Published:** 2021-05-31

**Authors:** Saravanan Rajendrasozhan, Essam N Ads, Amr S Abouzied, Jamal R Humaidi

**Affiliations:** 1 Department of Chemistry, College of Sciences, University of Haail, Haail 55476, Kingdom of Saudi Arabia; 2 Faculty of Science, Zagazig University, El Mansoura 35522, Egypt; 3 Department of Pharmaceutical Chemistry, College of Pharmacy, University of Hail, Hail 55476, Kingdom of Saudi Arabia; 4 Department of Pharmaceutical Chemistry, National Organization for Drug Control and Research, Giza 35521, Egypt

**Keywords:** Anticancer, cytotoxic, phytochemicals, natural remedies, Zizyphus spina-christi

## Abstract

Zizyphus spina-christi (Rhamnaceae family) is an edible plant used in folk medicine. Therefore, it is of interest to report the cytotoxic effects of Z. spina-christi bark crude extract on human cell lines. Crude ethanol extract of Z. spina-christi bark was fractionated with increasing polarity (diethyl ether, chloroform, ethyl acetate and butanol fractions). The fractions were examined for their cytotoxicity against human colon cancer (HCT-116 and CACO-2), cervical cancer (HeLa and HEp-2), lung carcinoma (A-549), hepatocellular carcinoma (HepG-2), breast cancer (MCF-7) and prostate cancer (PC-3) cell lines using viability assay. Diethyl ether fraction of Z. spina-christi showed the highest cytotoxic effects among the four extracts of Z. spina-christi. The IC50 of diethyl ether fraction was 7.14, 11.2, 11.6, 15.4, 39.8, 42.2, 84.2 and 153.8 µg/ml on HepG-2, A-549, CACO-2, HCT-116, MCF-7, PC-3, HeLa, and HEp-2 cell lines, respectively. Data shows that the diethyl ether fraction of Z. spina-christi showed effective cytotoxic effects in colon, lung and hepatocellular cancer cell lines.

## Background:

Resistance to chemotherapeutic agents, and adverse side effects of radiation are problems in clinical cancer treatment [1]. Drugs inspired by the natural products constitute many approved drugs for cancer as many as 247 drugs approved from 1981 to 2019 [2]. Therefore, it is of interest to report the cytotoxic effects of Z. spina-christi bark crude extract on human cell lines. It is commonly grown in desert areas with deficient rainfall, especially in the middle-east [3]. Z. spina-christi possesses significant amounts of diverse phytochemicals, most importantly flavonoids, saponins, tannins and triterpenes [4]. The bark of Z. spina-christi has potent biological activities such as antinociceptive [5], antidiarrheal [6] and antimicrobial [7] activities. The preliminary studies on the leaves of Z. spina-christi showed the potential cytotoxic effects against Hela and MDA-MB-468 tumor cells [8]. We have reported the cytotoxic effects of the Z. spina-christi stem bark extract in vitro [9]. Phytochemical analysis of stem bark of Z. spina-christi indicates the presence of tannins, flavonoids, terpernoids, saponin glycosides and alkaloids [9]. Moreover, in vivo studies in rats showed that Z. spina-christi could inhibit the early stage of colon carcinogenesis [10]. However, investigations on the cytotoxic activity of Z. spina-christi were limited. Therefore, it is of interest to document the in vitro cytotoxicity analysis of Zizyphus spina-christi stem bark extract on human cancer cell lines.

## Materials and methods:

### Chemicals:

Ethanol, diethyl ether, chloroform, ethyl acetate, butanol, dimethyl sulfoxide (DMSO), crystal violet and trypan blue dye were purchased from Sigma (St. Louis, MO, USA). Fetal bovine serum, Dulbecco's Modified Eagle Medium (DMEM), HEPES (N-(2-Hydroxyethyl) piperazine-N'-(2-ethanesulfonic acid)) buffer solution, L-glutamine, gentamycin, trypsin-EDTA were purchased from Lonza, Basel, Switzerland. All other chemicals and solvents used in this study were of analytical grade.

### Fractionation of Z. spina-christi bark extract:

Fresh bark of the Z. spina-christi was collected from the Hail region, Saudi Arabia. The plant was identified by Dr. Sherif Sayed Sharawy and the specimen was deposited in the Herbarium, Department of Biology, College of Sciences, University of Hail. The air-dried bark of Z. spina-christi was repeatedly extracted (3 times) at room temperature with absolute ethanol by percolation till complete exhaustion. The ethanol extract was filtered and solvents were evaporated under reduced pressure. The ethanol extract was then fractionated by successive extraction with the solvents of increasing polarity viz. diethyl ether, chloroform, ethyl acetate and n-butanol. The fractions were filtrated, and solvents were evaporated under reduced pressure.

### Mammalian cell lines:

CACO-2 cells (human intestinal carcinoma ATCC® HTB-37™), HCT-116 cells (human colon carcinoma ATCC® CCL-247™), PC-3 cells (human prostate carcinoma ATCC® CRL-1435™), HeLa cells (human cervical carcinoma ATCC® CCL-2™), A-549 cells (lung carcinoma ATCC® CCL-185™), HepG-2 (hepatocellular carcinoma ATCC® HB-8065™), MCF-7 (breast cancer cell line ATCC® HTB-22™) and HEp-2 cells (cervical carcinoma ATCC® CCL-23™) were purchased from American Type Culture Collection (ATCC), USA.

### Cell line propagation:

The cells were propagated in DMEM supplemented with 10% heat-inactivated fetal bovine serum, 1% L-glutamine, HEPES buffer and 50µg/ml gentamycin. All cells will be maintained at 37°C in a humidified atmosphere with 5% CO_2_ and were sub-cultured two times a week.

### Cytotoxicity evaluation using viability assay:

For cytotoxicity assay, the cells were incubated with different concentrations of the fractions of Z. spina-christi bark extract for 48 h. After the incubation period, the viable cells yield will be determined by the colorimetric method [11]. All results were corrected for background absorbance detected in wells without added stain. Treated samples were compared with the cell control in the absence of the Z. spina-christi fractions. All experiments were carried out in triplicate. The cell cytotoxic effects of Z. spina-christi fractions were calculated. The optical density was measured (SunRise microplate reader, TECAN, Inc, USA) to determine the number of viable cells and the percentage of viability was calculated as [(ODt/ODc)] x 100% where ODt is the mean optical density of treated wells (that was treated with Z. spina-christi fractions) and ODc is the mean optical density of untreated control wells. The relation between surviving cells and drug concentration is plotted to get the survival curve of each tumor cell line after treatment with the specified Z. spina-christi fraction.

### The half-maximal inhibitory concentration (IC50):

The concentration of Z. spina-christi fractions required to cause cytotoxic effects in 50% of cancer cells (LD50) will be estimated from graphic plots of the dose-response curve for each concentration using Graphpad Prism software (San Diego, CA. USA).

### Statistical analysis:

The obtained results were analyzed using SPSS, IBM, Armonk, New York, United States to obtain the standard deviation (SD). The results were expressed as mean ± SD.

## Results:

### Cytotoxicity of Z. spina-christi against colon cancer cell lines (CACO-2 and HCT-116):

**Figure 1:**
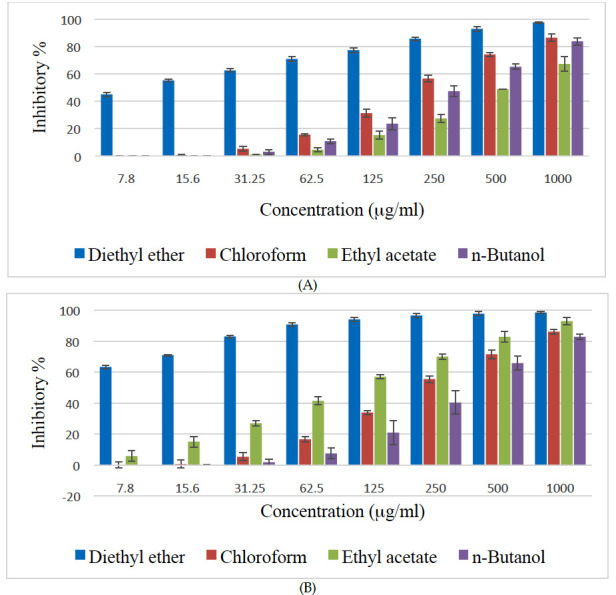
Cytotoxic effects of various fractions of Z. spina-christi bark against human colon carcinoma cell lines (A) CACO-2 and (B)HCT- 116.

The control cells showed high proliferation that has been taken as 100% viability (0% cytotoxicity). As shown in Figure 1, the diethyl ether fraction of Z. spina-christi extract showed the highest dose-dependent (7.8 - 1000 mg/ml) cytotoxic effect against CACO-2 and HCT-116 cell lines. This fraction showed more than 90% inhibitory activity against HCT-116 from the concentration of 62.5 mg/ml. Other fractions showed considerable effect only at concentrations more than 31.25 mg/ml. The second-highest cytotoxic activity was shown by the chloroform extract against CACO-1, and by the ethyl acetate fraction against HCT-116.

### Cytotoxicity of Z. spina-christi against cervical cancer cell lines (HeLa and HEp-2):

**Figure 2:**
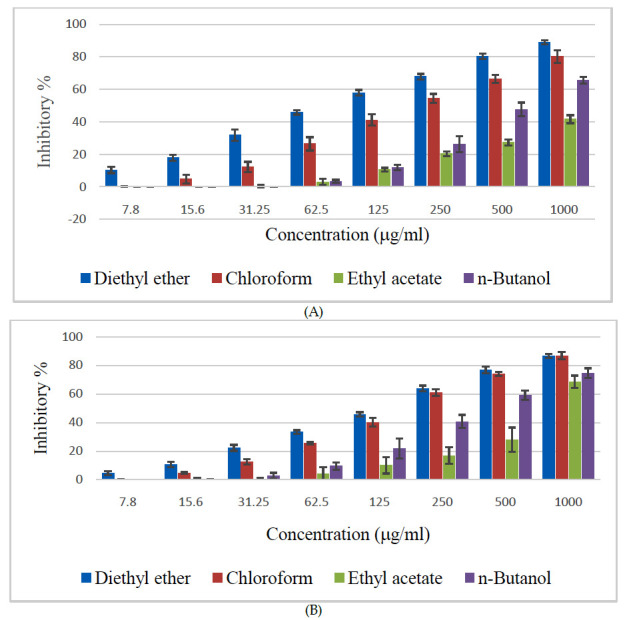
Cytotoxic effects of various fractions of Z. spina-christi bark extract against human cervical carcinoma cell lines (A) HeLa and (B)HEp-2.

Diethyl ether fraction of Z. spina-christi extract showed the highest cytotoxic effects against the cervical cancer cell lines at all the tested concentrations in a dose-dependent manner (7.8 - 1000 mg/ml) (Figure 2). Other fractions also showed considerable effects at higher concentrations. The chloroform fraction showed the second-highest cytotoxicity effect against HeLa and HEp-2. In addition, chloroform fraction exhibited a comparable effect as that of diethyl ether fraction against HEp-2 at higher concentrations.

### Cytotoxicity of Z. spina-christi against lung cancer cell lines (A-549):

**Figure 3:**
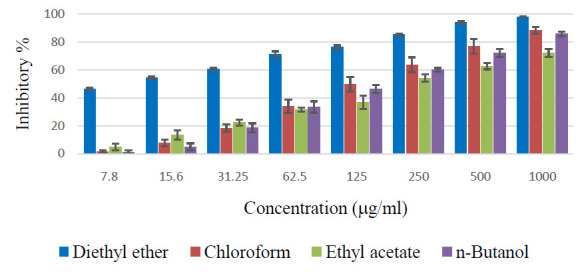
Cytotoxic effects of various fractions of Z. spina-christi bark extract against human lung cancer cell lines (A-549).

As shown in Figure 3, the diethyl ether fraction of Z. spina-christi extract showed a potent cytotoxic effect against the A-549 cell line even at low concentration (>40% of inhibitory activity at 7.8 mg/ml). All the fractions showed considerable cytotoxic activity at higher concentrations. Among the four fractions, chloroform fraction showed the second-highest activity, especially from the concentrations >62.5 mg/ml.

### Cytotoxicity of Z. spina-christi against hepatocarcimoma cell lines (HepG-2):

**Figure 4:**
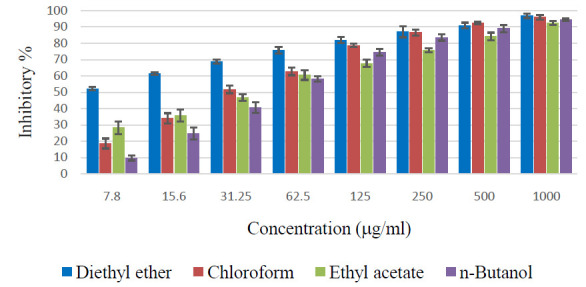
Cytotoxic effects of various fractions of Z. spina-christi bark extract against human hepatocarcinoma cell lines (HepG-2).

The diethyl ether fraction of Z. spina-christi extract showed a potent cytotoxic effect (>50% inhibition) against HepG-2 cell line from the concentration of 7.8 mg/ml (Figure 4). All the fractions showed a dose-dependent increase in cytotoxicity. Although the ethyl acetate fraction is effective than the chloroform fraction at low concentration (7.8 mg/ml), overall, the chloroform extract showed the second-highest cytotoxic effect.

### Cytotoxicity of Z. spina-christi against breast cancer cell lines (MCF-7):

**Figure 5:**
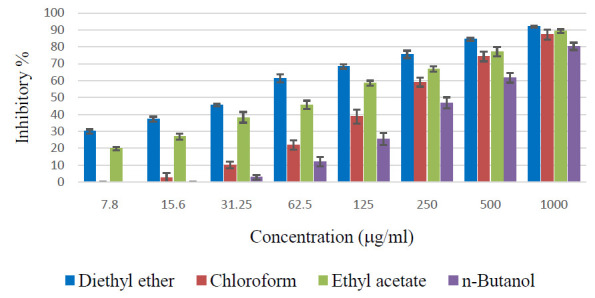
Cytotoxic effects of various fractions of Z. spina-christi bark extract against human hepatocarcinoma cell lines (HepG-2).

The diethyl ether and ethyl acetate fractions of Z. spina-christi extract showed a cytotoxic effect against the MCF-7 cell line from the concentration of 7.8 mg/ml. This fraction showed a dosedependent increase in the cytotoxicity with about 80% of cytotoxic effect at 1000 mg/ml (Figure 5). The chloroform and butanol fractions showed cytotoxic effects from the concentration of 15.6 and 31.2 mg/ml, respectively.

### Cytotoxicity of Z. spina-christi against prostate cancer cell lines (PC-3):

**Figure 6:**
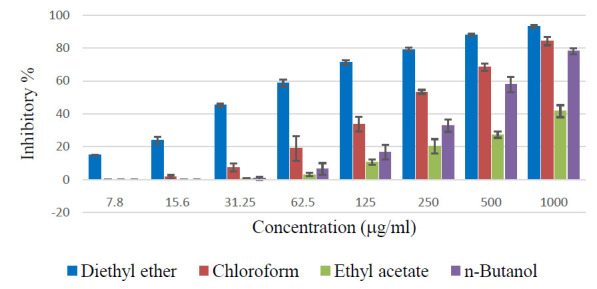
Cytotoxic effects of various fractions of Z. spina-christi bark extract against human prostate carcinoma cell lines (PC-3).

The diethyl ether fraction of Z. spina-christi extract showed a potent cytotoxic effect against PC-3 cell line from the concentration of 7.8 mg/ml. This fraction showed a dose-dependent increase in the cytotoxicity with more than 90% of cytotoxic effect at 1000 mg/ml (Figure 6). The chloroform fraction showed considerable cytotoxic effect from the concentration of 15.6 mg/ml, whereas other fractions showed considerable effects only at concentrations of more than 62.5 mg/ml.

### IC50 of fractions of Z. spina-christi extract against various cell lines:

The IC50 values of diethyl ether fraction is lower than other fractions against all the tested cell lines (Table 1 - see PDF). The IC50 values of diethyl ether fraction are less than 10 mg/ml against HepG-2, less than 20 mg/ml against A-549, CACO-2 and HCT-116, less than 50 mg/ml against MCF-7 and PC-2, and more than 100 mg/ml against HEp-2. The diethyl ether fraction is highly active against HepG-2 (IC50=7.1 mg/ml) and least active against HEp-2 (IC50=153.8 mg/ml). The cytotoxic potency of diethyl ether fraction against various cell lines is descended in the following order: HepG-2 > A-549 > CACO-2 > HCT-116 > MCF-7 > PC-3 > HeLa > HEp-2. Ethyl acetate fraction showed cytotoxic effects with the IC50 value of less than 100 mg/ml against A-549, HepG-2, HCT-116, whereas chloroform and butanol fractions showed cytotoxic effects with the IC50 value less than 100 mg/ml only against HepG-2.

## Discussion:

Identification of new chemical entities through the biological activity-guided fractionation of plant materials is a novel approach to integrated drug discovery [12]. The phytochemical screening of ethanolic extract of Z. spina-christi bark showed the presence of tannins, flavonoids, terpenoids, saponin glycosides and alkaloids in the stem bark [9]. Because of the existence of bioactive compounds in the ethanolic extract, a preliminary study was conducted to elucidate the cytotoxic effect of Z. spina-christi bark extract using two human cancer cell lines, HCT-116 and MCH-7 [9]. Once the extract is found to be biologically active, the next recommended step is to proceed with fractionation of the extract using solvents with different polarities [13]. To further understand the cytotoxic effect of Z. spina-christi bark, four fractions were isolated from the ethanolic extract of Z. spina-christi bark based on polarity and tested for their cytotoxic effects against eight different human cancer cell lines.

Diethyl ether fraction of Z. spina-christi bark extract exhibited remarkable cytotoxic activity against the cell lines (with values of IC50 7.1, 11.3, 11.6, 15.4, 39.8, 42.2, 84.2 and 153.8 µg/ml against HepG-2, A-549, CACO-2, HCT-116, MCF-7, PC-3, HeLa, and HEp-2 cell lines, respectively). The fraction with low polarity (diethyl ether fraction) showed the highest cytotoxic effects than the fractions with high polarity (chloroform, ethyl acetate and butanol fractions). Comparing the IC50 of all the fractions against cancer cell lines revealed that diethyl ether extract has multi-fold potent cytotoxic activity compared to the second-highly active fraction (1 fold to 18 fold). The lower IC50 values represent the greater cytotoxic activity, whereas the higher the IC50 values represent the lower cytotoxic activity. It was reported that tradecene, hexadecanoic acid, ethyl ester (ethyl palmitate) and ethyl linoleate are found only in diethyl ether fraction, not in other fractions of Z. spina-christi bark extract [14]. One or a few of the unique bioactive compounds present in the diethyl ether fraction may be responsible for their potent cytotoxic effects compared to other fractions. Hexadecanoic acid (palmitic acid) has a potent cytotoxic effect against human leukemic cells in vitro and antitumor activity in vivo. It is reported that hexadecanoic acid exhibited the cytotoxic effect by selectively inhibiting DNA topoisomerase I in the tumor cells [15]. Docking study confirmed the interaction between hexadecanoic acid and DNA topoisomerase I. Thus, the presence of hexadecanoic acid and ethyl ester (ethyl palmitate) in the diethyl ether fraction may be responsible for preventing cell proliferation. Chloroform fraction of Z. spina-christi bark extract exhibited the second-highest cytotoxic activity (after diethyl ether fraction) against the five cell lines (with the IC50 of 217.9, 208.2, 184.0, 29.7 and 228.2 µg/ml against CACO-2, HeLa, HEp-2, HepG-2 and PC-3, respectively). Betulin (a triterpene) was present in chloroform extract in large quantities as compared with other fractions. Betulin has shown anticancer and chemopreventive potentials against several established cancer cell lines, as well as primary tumor cell cultures in vitro and in vivo [16]. Betulin treatment can induce the expression of some cellular proteins indirectly involved in apoptosis. Betulin has the potential to selectively inhibit the activity of DNA topoisomerases II, without any influence on DNA topoisomerases I. Betulinic acid, a derivative of botulin, is a wellknown inhibitor of other cancerous tumors, including human colon carcinoma and human prostate adenocarcinoma [17]. Either ethyl acetate fraction (against CACO-2, HeLa, HEp-2 and PC-3 cell lines) or butanol fraction (against HCT-116, A-549, HepG-2 and MCF-7 cell lines) showed the least cytotoxic activity among the four fractions of Z. spina-christi bark extract. On the other hand, ethyl acetate fraction showed a considerable cytotoxic effect against HCT-116, A-549 and MCF-7 than the chloroform and butanol fractions (but less effective than diethyl ether fraction). This selectivity of ethyl acetate fraction should be considered for further studies. When comparing the effect of highly-active diethyl ether fraction on various human cancer cell lines, this fraction showed potent cytotoxic activity against intestinal and colon (CACO-2 and HCT-116), lung (A-549) and liver (HepG-2) cancer cell lines. Interestingly, diethyl ether fraction showed a better cytotoxic effect against CACO-2 than the standard drug, vinblastine sulfate. As colon cancer is the most common form of fatal cancer worldwide, further isolation of bioactive from diethyl ether fraction is warranted for potential drug development.

## Conclusion:

The fractions of ethanolic extract of Z. spina-christi bark showed cytotoxic effects on various human cancer cell lines (colon, cervical, lung, liver, breast and prostate cancer cell lines). The cytotoxic effects of fractions against CACO-2, HeLa, HEp-2, HepG-2 and PC-3 are in the order of diethyl ether >> chloroform > butanol > ethyl acetate. The cytotoxic effect of fractions against HCT-116, A-549 and MCF-7 are in the order of diethyl ether >> ethyl acetate > chloroform > butanol. The diethyl ether fraction of ethanolic extract of Z. spina-christi showed the highest activity compared to other highly-polar fractions (chloroform, ethyl acetate, and n-butanol fractions) for further consideration in cancer drug discovery.
